# Impact of major illnesses and geographic regions on do-not-resuscitate rate and its potential cost savings in Taiwan

**DOI:** 10.1371/journal.pone.0222320

**Published:** 2019-09-12

**Authors:** Ming-Tai Cheng, Fuh-Yuan Shih, Chu-Lin Tsai, Hung-Bin Tsai, Daniel Fu-Chang Tsai, Cheng-Chung Fang

**Affiliations:** 1 Department of Emergency Medicine, National Taiwan University Hospital, Taipei, Taiwan; 2 Department of Internal Medicine, National Taiwan University Hospital, Taipei, Taiwan; 3 Department of Medical Research, National Taiwan University Hospital, Taipei, Taiwan; 4 Graduate Institute of Medical Education and Bioethics, National Taiwan University College of Medicine, Taipei, Taiwan; Hong Kong Polytechnic University, HONG KONG

## Abstract

**Background/Purpose:**

Do-not-resuscitate (DNR) is a legal order that demonstrates a patient’s will to avoid further suffering from advanced treatment at the end of life. The concept of palliative care is increasingly accepted, but the impacts of different major illnesses, geographic regions, and health expenses on DNR rates remain unclear.

**Methods:**

This study utilized the two-million National Health Insurance (NHI) Research Database to examine the percentage of DNR rates among all deaths in hospitals from 2001 to 2011. DNR in the study was defined as no resuscitation before death in hospitals. Death records were extracted from the database and correlated with healthcare information. Descriptive statistics were compiled to examine the relationships between DNR rates and variables including major illnesses, geographic regions, and NHI spending.

**Results:**

A total of 126,390 death records were extracted from the database for analysis. Among cancer-related deaths, pancreatic cancer patients had the highest DNR rate (86.99%) and esophageal cancer patients had the lowest DNR rate (71.62%). The higher DNR rate among cancer-only patients (79.53%) decreased with concomitant dialysis (66.07%) or ventilator use (57.85%). The lower DNR rates in patients with either chronic dialysis (51.27%) or ventilator use (59.10%) increased when patients experienced these two conditions concomitantly (61.31%). Although DNR rates have consistently increased over time across all regions of Taiwan, a persistent disparity was noted between the East and the South (76.89% vs. 70.78% in 2011, *p* < 0.01). After adjusting for potential confounders, DNR patients had significantly lower NHI spending one year prior to death ($67,553), compared with non-DNR patients.

**Conclusion:**

Our study found that DNR rates varied across cancer types and decreased in cancer patients with concomitant chronic dialysis or ventilator use. Disparities in DNR rates were evident across geographic regions in Taiwan. A wider adoption of the DNR policy may achieve substantial savings in health expenses and improve patients’ quality of life.

## Introduction

Do-not-resuscitate (DNR) is a legal order that demonstrates a patient’s will to avoid receiving cardiopulmonary resuscitation (CPR) and life-sustaining treatment (LST) at the end of life. In terminally ill patients, CPR and LST not only result in wastage of medical resources, but they also increase patients’ suffering. Hospice palliative care, instead of CPR and LST, provides mitigatory and supportive medical care to relieve physical, mental, and spiritual agonies and improve patients’ quality of life.

The promotion and legislation of palliative care in Taiwan began with the announcement of the regulations governing palliative care on June 7, 2000. Taiwan was the first Asian country to pass such a law. The regulations were revised on December 11, 2002 and January 26, 2011 to simplify the procedures of removing life support and terminating CPR and to increase the clarity of such measures. The DNR rate increased gradually over time in Taiwan, rising from 60.7% in 2001 to 73.4% in 2011[1]. The factors associated with DNR rates in Taiwan were major illnesses, gender, age, hospital size, and marital status. Among patients with major illnesses, cancer patients had the highest DNR rate, and long-term dialysis patients had the lowest DNR rate, compared to patients with non-major illnesses[1]. However, the differences in DNR rates among cancer subtypes remain unclear. Patients may suffer from more than one major illness, and the concomitant major illness may affect the DNR rate. This topic thus requires further study.

Regional culture has been shown to play an important role in end-of-life decision-making because it affects patients, their families, and the healthcare providers[2] living or working in that area. Although Taiwan is a small island, there are still many geographic, climatic, and cultural differences across the regions. We therefore also explored whether any geographic differences exist in the DNR rates in the current study.

In addition, good palliative care can potentially conserve and allocate resources to other patients who are in dire need in the health care system[3]. Studies have shown that implementation of palliative care decreased length of hospital stays significantly[3, 4]. No nationwide study has qualified the cost differences between DNR and non-DNR patients in Taiwan; therefore, we also aimed to answer this important question in the current study.

## Methods

The National Health Insurance (NHI) program in Taiwan commenced in 1995 and covers over 98% of the total population of 23 million inhabitants[5]. The National Health Research Institutes in Taiwan used the NHI claims database as the mother population and generated a simple random sampling of two million individuals (two-million NHI Research Database) to match the mother population in distribution of age, gender, and averaged insured amount. The medical information (outpatient, hospitalization, and affiliated pharmacy) of each individual was extracted. This research was conducted after obtaining approval from the Research Ethics Committee at National Taiwan University Hospital (NTUH-REC No.:201312068W).

In this study, we analyzed data from the two-million NHI Research Database from 2001 to 2011. We used the National Death Registry in Taiwan to identify deceased patients and the National Cancer Registry to identify cancer patients. The location of the patient’s death (in hospital or at home) was obtained from the death certificate. For patients who died in hospital, the last NHI-claims data file (hospitalization and emergency department visit) was used to identify whether the patient received cardiac massage, defibrillation, an endotracheal tube, tracheostomy, ventilator treatment, or other treatment using life support devices. However, the data in this claims-based study did not contain direct information on DNR orders; thus, patients who did not receive these life support measures or devices were considered to have DNR orders. Therefore, DNR in this study was technically defined as no resuscitation before patients’ death.

As mentioned above, we defined individuals who received cardiac massage (code: 47029C) or defibrillation (code: 47028C) before death during their last hospitalization or emergency department visit as non-DNR patients. Remaining cases were further analyzed to determine if they received ventilation support with any of the following codes: endotracheal tube (code: 47031C), tracheostomy (code: 56003C), ventilator treatment (code: 57001B), or high frequency ventilator treatment (code: 57029C). Patients who received ventilation support were also defined as non-DNR patients. In our NHI coding, patients with long-term ventilator dependence (including “home ventilator use”) do not use the aforementioned ventilator codes.

The NHI defines several diseases as “major illnesses” to exempt co-payment and includes individuals with cancers, congenital diseases, autoimmune diseases, major trauma and burns, psychosis, ventilator dependence, chronic dialysis, and organ transplantation. We categorized major illnesses into four groups: cancer (CA), chronic dialysis (CD), ventilator dependence (VD), and other major illness (OT).

To examine DNR rates based on region, we divided Taiwan’s cities and counties into North, Central, South, and East regions. The North region consisted of Taipei City, New Taipei City, Keelung City, Yilan County, Taoyuan City, Hsinchu County, Hsinchu City, Kinmen County, and Lienchiang County. The Central region consisted of Miaoli County, Taichung City, Nantou County, Changhua County, and Yunlin County. The South region consisted of Chiayi County, Chiayi City, Tainan City, Kaohsiung City, Pingtung County, and Penghu County. The East region consisted of Hualien County and Taitung County. These geographic divisions were made based on the Jhonggang River (separating Hsinchu and Miaoli in the Qing Dynasty), the Tropic of Cancer (separating the subtropics and tropics), and the Central Mountain Range (separating East and West Taiwan). Offshore regions were included in their six respective NHI regions, i.e., Kinmen County and Lienchiang County were allocated to the North Region, and Penghu County was allocated to the South region.

Patients were divided into DNR and non-DNR groups for analysis of NHI spending. Using data collected from the NHI database, patients’ inpatient and outpatient NHI spending one year prior to death was calculated. The average numbers of points used by the cases in each group were calculated. Each point value (and the locations of the hospital visited by the patients) may vary with time; however, for easy comparison, NT$1 was counted as one point.

Descriptive statistics were used to examine the relationships between DNR rates and variables including major illnesses, geographic regions, and NHI spending. Specifically, one-way analysis of variance (ANOVA) was applied to compare DNR rates between geographic regions, and pairwise comparisons were performed using Tukey’s method. Student’s t-test was used to test for differences in NHI spending between DNR and non-DNR groups. Mixed-effect models were used to examine the effect of major illness and geographic regions on DNR rates using the GLIMMIX (Generalized Linear Mix Model) procedure (SAS ® version 9.4 software) with patients clustered within regions. All *P* values are two-sided, with *P*<0.05 considered statistically significant.

## Results

**Fig 1:**
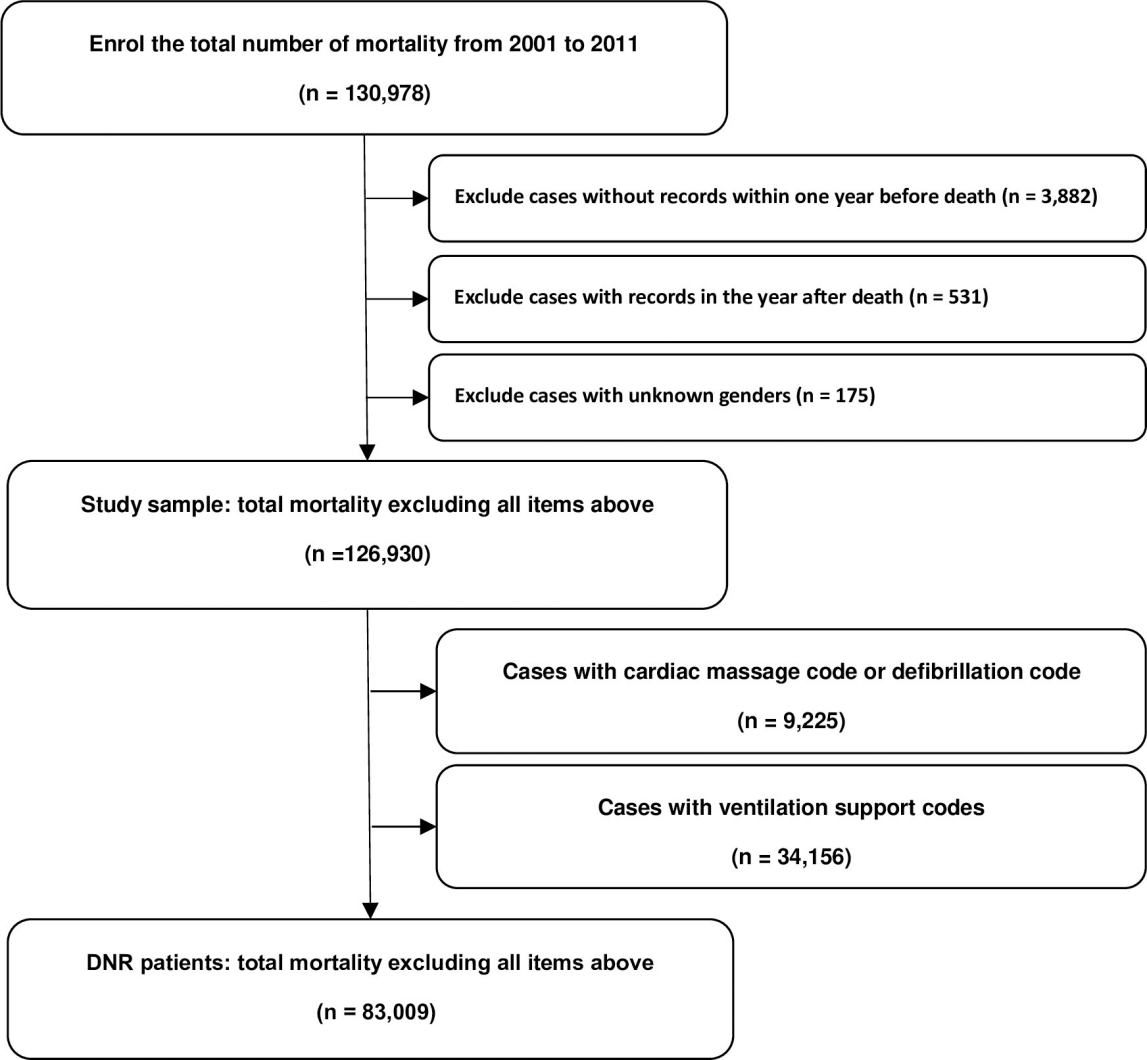
The sample selection process used in the study.

The study population comprised 130,978 deceased individuals. After excluding cases with no insurance record one year before death, cases with insurance records obtained after death, and cases with unknown gender, 126,390 cases underwent further analysis (Fig 1). After we further excluded patients who received cardiac massage, defibrillation and ventilator support before death, 83,009 cases were considered as DNR patients. The total DNR rate in this study was 65.68%. There were 38,656 cancer-related deaths between 2001 and 2011 (Table 1). Among these cases, 30,374 (78.58%) did not receive the life support measures defined in the Methods section of this study and were therefore considered DNR patients. There were 87,734 cases who died from causes other than cancer, and 52,635 of them (59.99%) met the DNR criteria.

**Table 1:** Adjusted odds ratios (aOR) of do-not-resuscitate (DNR) rates in different type of cancer patients.

Type of malignancy	Total death	DNR No.	DNR%	aOR**	95% C.I.
Pancreatic cancer	584	508	86.99	2.40	(1.77–3.24) *
Gastric cancer	1,640	1,322	80.61	1.33	(1.14–1.55) *
Head & neck cancers	1,239	992	80.06	1.31	(1.10–1.57) *
Liver and intrahepatic bile duct cancers	4,117	3,321	80.67	1.28	(1.16–1.42) *
Trachea, bronchus and lung cancers	4,158	3,269	78.62	1.12	(1.02–1.24) *
Hematological malignancies	1,067	783	73.38	0.78	(0.66–0.93) *
Esophageal cancer	740	530	71.62	0.78	(0.65–0.95) *
Colorectal cancers	2,604	2,072	79.57	1.14	(1.00–1.29)
Breast cancers	1,300	1,063	81.77	1.07	(0.89–1.29)
Prostatic cancer	1,329	989	74.42	1.07	(0.89–1.28)
Cervical cancer	656	504	76.83	0.90	(0.72–1.13)
Other cancers	19,222	15,021	78.14	Ref.	
Total	38,656	30,374	78.58		

* Statistically significant

** Controlling for age, gender, calendar year and geographic areas by the GLIMMIX procedure.

*** ICD-9-code of each cancer: Pancreatic cancer (ICD-9-code:157), Gastric cancer (ICD-9-code:151), Head & neck cancers (ICD-9-code:140–141 143–146 148–149), Liver and intrahepatic bile duct cancers (ICD-9-code:155), Colorectal cancers (ICD-9-code:153–154), Trachea, bronchus and lung cancers (ICD-9-code:162), Hematological malignancies (ICD-9-code:200–208), Esophageal cancer (ICD-9-code:150), Breast cancers (ICD-9-code:174–175), Prostatic cancer (ICD-9-code:185), Cervical cancer (ICD-9-code:179–180).

**Fig 2:**
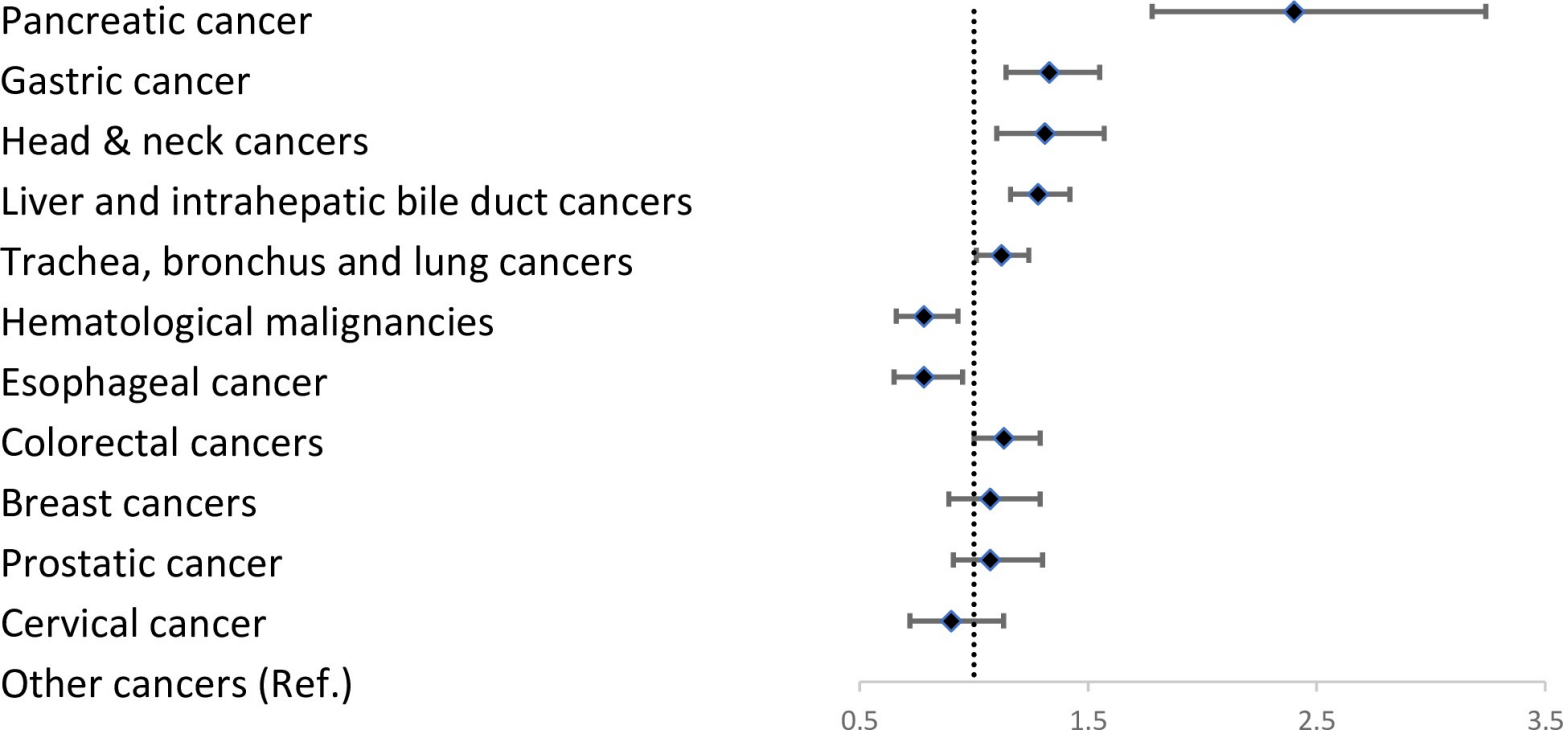
Forest plot: Adjusted odds ratios (aOR) of do-not-resuscitate (DNR) rates by type of cancer.

Table 1 and Fig 2 show the types of cancer and relevant DNR rates. The table shows that patients with pancreatic cancer had the highest DNR rate (86.99%, adjusted odds ratio [aOR]: 2.4, 95% confidence interval [CI]: 1.77–3.24), whereas those with esophageal cancer had the lowest DNR rate (71.6%, aOR: 0.78, 95% CI: 0.65–0.95), compared to patients with other cancer types with DNR rate (78.14%).

**Fig 3:**
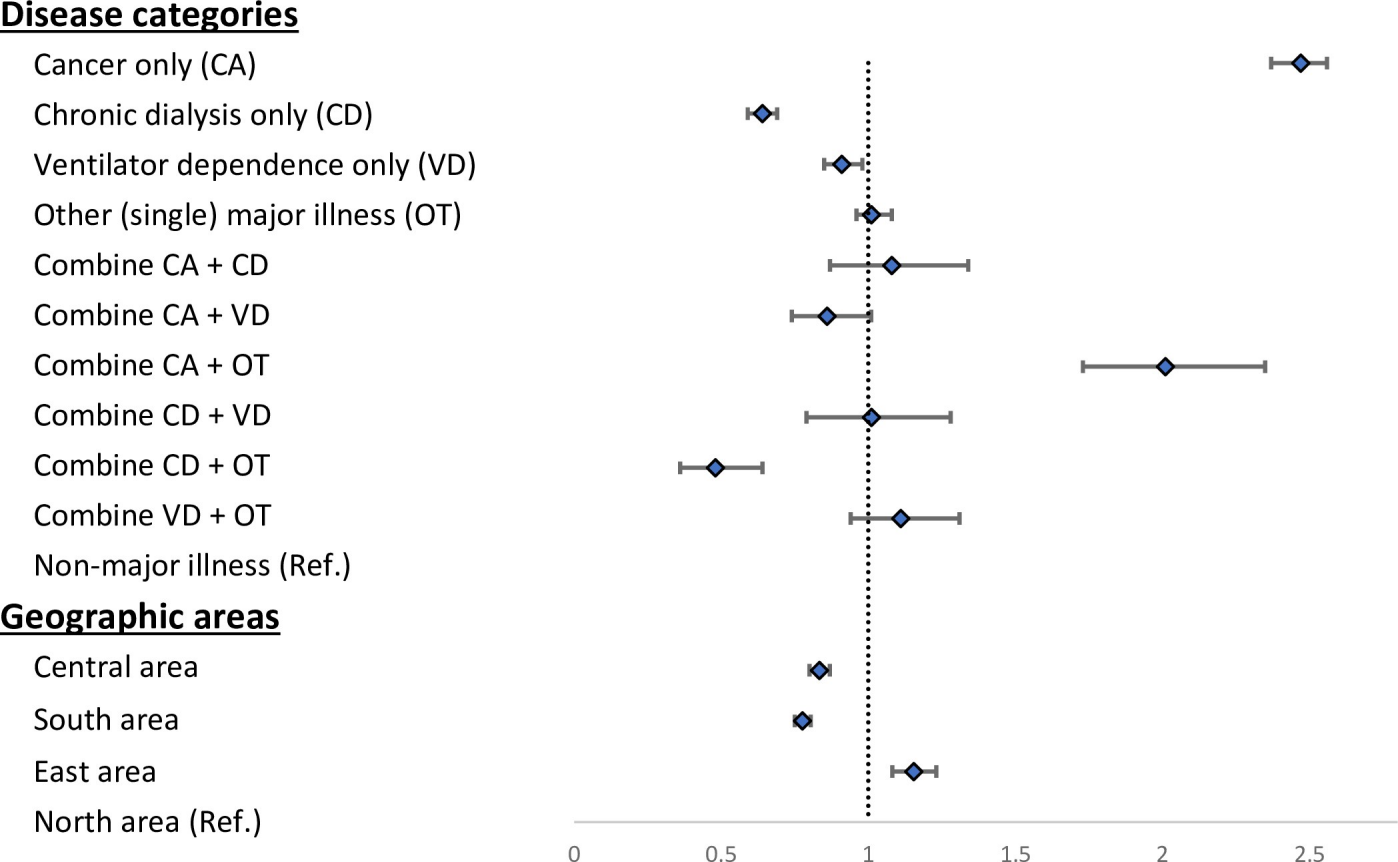
Forest plot: Adjusted odds ratios (aOR) of do-not-resuscitate (DNR) rates among different and combined major illnesses and geographic areas.

Concomitant major illnesses may affect the DNR rate. Table 2 and Fig 3 show the results of major illness combinations. Compared with patients without a major illness, cancer-only patients had the highest DNR rate (79.53%, aOR: 2.47, 95% CI: 2.37–2.56), whereas patients with chronic dialysis combined with another major illness had the lowest DNR rate (46.88%, aOR: 0.48, 95% CI: 0.36–0.64). The higher DNR rate among cancer patients decreased when these patients had concomitant dialysis (66.07%) or ventilator use (57.85%). Compared to non-major illness cases, the chronic dialysis-only group (aOR: 0.64, 95% CI: 0.59–0.69) and the ventilator-dependent-only group (aOR: 0.91, 95% CI: 0.85–0.98) had lower DNR rates.

**Table 2:** Adjusted odds ratios (aOR) of do-not-resuscitate (DNR) rates among different and combined major illnesses and geographic areas.

	Total Death	DNR No.	DNR%	aOR**	95% C.I.
**Disease categories**					
Cancer only (CA)	35,439	28,186	79.53	2.47	(2.37–2.56) *
Chronic dialysis only (CD)	4,521	2,318	51.27	0.64	(0.59–0.69) *
Ventilator dependence only (VD)	6,946	4,105	59.10	0.91	(0.85–0.98) *
Other (single) major illness (OT)	10,224	6,220	60.84	1.01	(0.96–1.08)
Combine CA + CD	731	483	66.07	1.08	(0.87–1.34)
Combine CA + VD	1,255	726	57.85	0.86	(0.74–1.01)
Combine CA + OT	1,612	1,247	77.36	2.01	(1.73–2.35) *
Combine CD + VD	579	355	61.31	1.01	(0.79–1.28)
Combine CD + OT	369	173	46.88	0.48	(0.36–0.64) *
Combine VD + OT	1,143	723	63.25	1.11	(0.94–1.31)
Non-major illness	63,242	38,258	60.49	Ref.	
**Geographic areas**					
Central area	26,502	17,029	64.26	0.83	(0.80–0.87) *
South area	47,123	29,237	62.04	0.78	(0.75–0.80) *
East area	8,644	6,194	71.66	1.15	(1.08–1.23) *
North area	54,110	36,844	68.09	Ref.	

*Statistically significant.

** Controlling for age, gender, calendar year and geographic areas by the GLIMMIX procedure.

***Combination over 2 groups of major illnesses were not list due to small sample sizes

**Fig 4:**
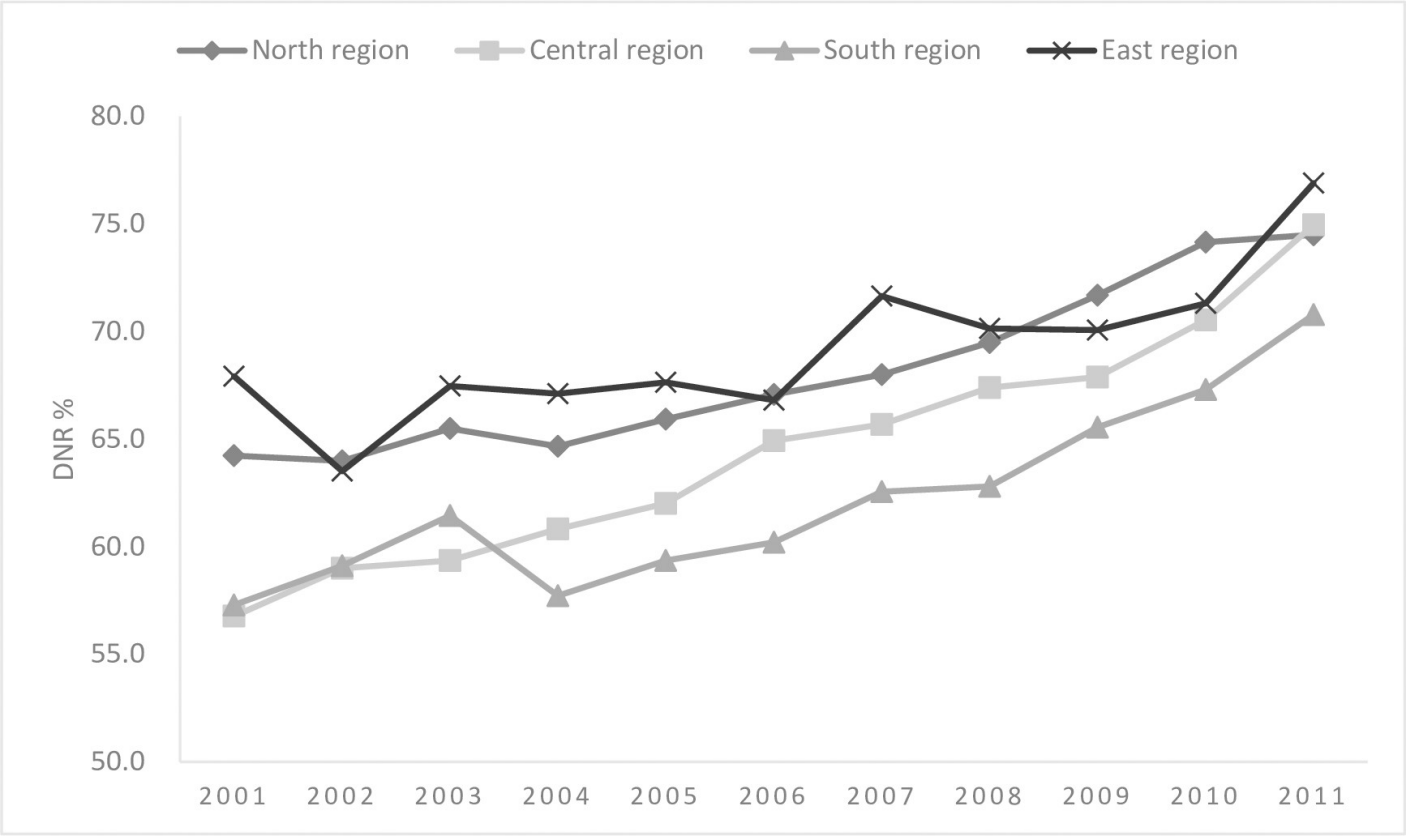
Geographic differences in do-not-resuscitate (DNR) rates in Taiwan.

Fig 4 illustrates the DNR rates of the different geographic regions between 2001 and 2011. Overall, the DNR rates were different across regions (*p* = 0.002). Although the DNR rates have consistently increased over time across all regions of Taiwan, persistent disparity was noted between the East and the South (76.89% vs. 70.77% in 2011, *p* < 0.01), and between the North and the South (74.49% vs. 70.77% in 2011, *p* < 0.05). The regional disparities persisted in multivariable analyses, as reported in Table 2. The results of multivariate analyses showed people in the East had the highest DNR rate (71.66%, aOR: 1.15, 95% CI: 1.08–1.23, vs. the North), while people in the South had the lowest DNR rate (64.02%, aOR: 0.78, 95% CI: 0.75–0.8 vs. the North).

**Fig 5:**
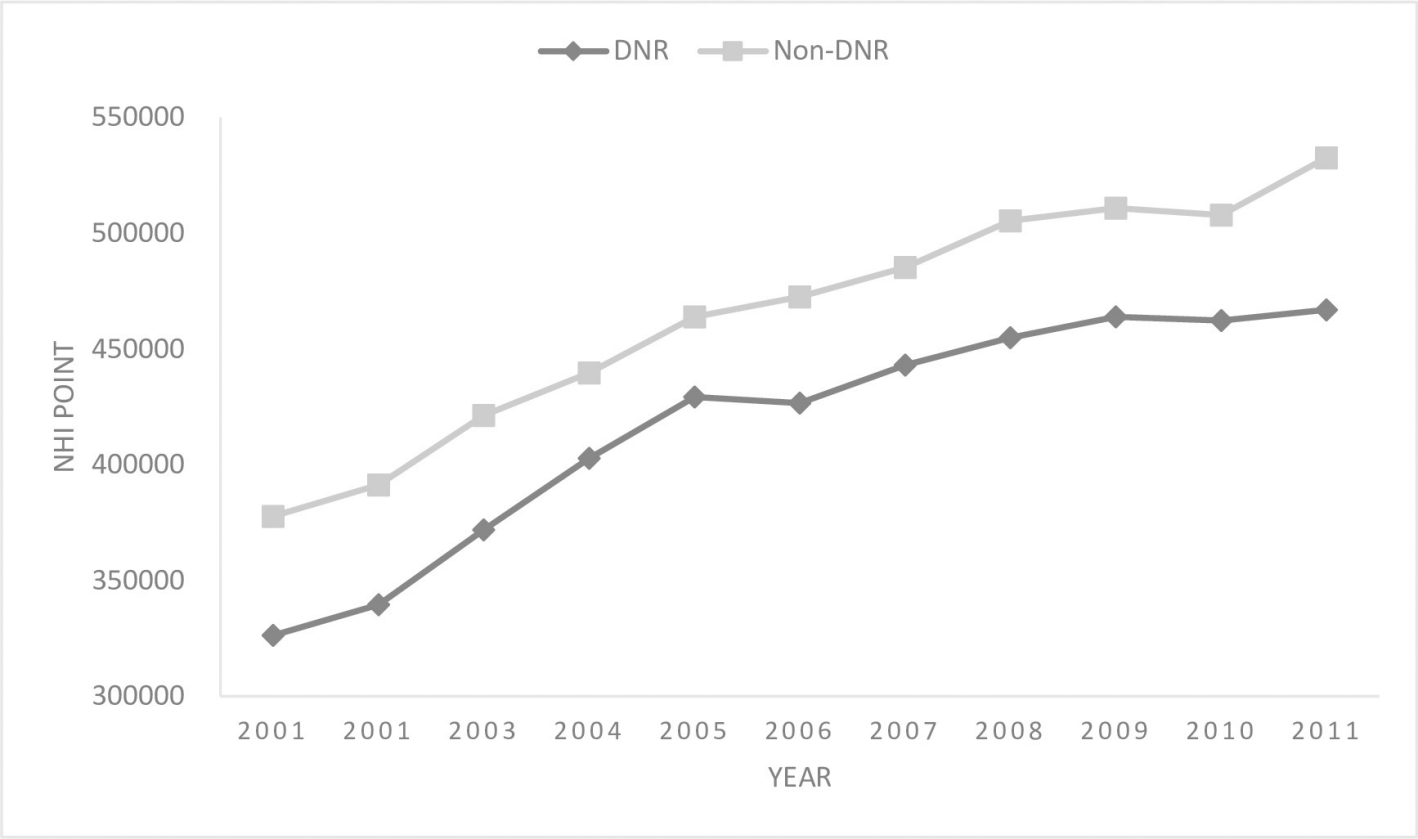
Average National Health Insurance (NHI) spending on do-not-resuscitate (DNR) and Non-DNR cases in Taiwan.

Fig 5 shows the calculated average NHI spending of the last year before death for each patient. Findings indicated that NHI spending in both DNR and non-DNR groups increased over time but was consistently higher for the non-DNR group than for the DNR group (*p* < 0.05). The minimal difference between the two groups was recorded in 2005 at NT$34,690 (7.48% of non-DNR group average spending in 2005), and the maximum was recorded in 2011 at NT$65,684 (12.33% of non-DNR group average spending in 2011). After controlling for factors including age, gender, major illness, calendar year and geographic areas, the estimated cost difference between the two groups widened slightly to NT$67,553 (Table 3). All major illnesses were associated with increased health expenditure, especially in patients with chronic dialysis and/or ventilator (Table 3).

**Table 3:** Adjusted* cost differences between DNR and Non-DNR groups.

	Estimate	P value	95% C.I.
**DNR group vs Non-DNR group**	-67,553	<0.0001	-72,769 to -62,337
Disease categories**			
Cancer only (CA)	264,049	<0.0001	258,107 to 269,992
Chronic dialysis only (CD)	661,040	<0.0001	647,350 to 674,731
Ventilator dependence only (VD)	792,906	<0.0001	781,276 to 804,537
Other (single) major illness (OT)	155,585	<0.0001	146,214 to 164,956
Combine CA + CD	737,088	<0.0001	700,438 to 773,738
Combine CA + VD	908,413	<0.0001	881,222 to 935,604
Combine CA + OT	287,429	<0.0001	263,733 to 311,126
Combine CD + VD	1,455,014	<0.0001	1,413,876 to 1,496,152
Combine CD + OT	765,853	<0.0001	717,054 to 814,651
Combine VD + OT	825,511	<0.0001	796,768 to 854,254
Non-major illness	Ref.		

* Controlling for age, gender, calendar year and geographic areas by the GLIMMIX procedure.

**Combination over 2 groups of major illnesses were not list due to small sample sizes.

## Discussion

Our study used nationwide data to show the differences in DNR rates between cancer types and combinations of major illnesses. We found that DNR rates varied across cancer types. However, DNR rates decreased among cancer patients with concomitant chronic dialysis or ventilator use. All of the above-mentioned variation in DNR can be viewed as variation in “highly aggressive care near death” because our definition of DNR is no resuscitation prior to death. We also demonstrated regional differences in DNR rates in Taiwan as well as spending differences between DNR and non-DNR patients.

Our previous study showed that cancer patients had a high DNR rate (78.58%, as also shown in Table 1)[1]. In Western countries, studies have shown that the inpatient DNR rates of cancer patients were around 86% in the US[6] and 88% in Australia[7], but promotion of the concept of palliative care is in its infancy in Asian countries[8]. A Korean study showed a 12% DNR rate in metastatic cancer patients with palliative chemotherapy in one hospital[9], while another hospital reported an 87% DNR rate in terminal cancer patients[10]. A three-month Japanese nationwide survey showed that 76% of terminal cancer patients in palliative care units had a DNR order[11]. However, all aforementioned studies were limited to specialized study groups and study periods, and few previous studies have analyzed the correlation between cancer types and DNR rates. The present study found different DNR rates for each cancer type, ranging from 71.62% to 86.99%. Pancreatic cancer patients had the highest DNR rate, which may reflect the grave prognosis in this group of cancer patients. However, esophageal cancer patients had the lowest DNR rate in our study. This finding could be explained by the fact that some of these patients received tracheostomies during esophageal cancer surgery, leading them to be defined as non-DNR patients in our study. Patients with hematological malignancies also had lower DNR rates among cancers. Patients with hematologic malignancy sometimes suffer from sudden onset sepsis which is difficult to predict in the prognosis. Therefore, hematologists may perform a time-limited trial, which results in using LST.

We were also interested in how combinations of major illnesses influenced DNR rates. Because of special regulations concerning major illnesses ratified in Taiwan, it was difficult to compare the DNR rates of major illnesses in Taiwan with those of other countries. As shown in Table 2, cancer patients’ DNR rates generally decreased when they suffered from another major illness concomitantly. This trend is not readily explained, as prognoses become less optimistic with increased medical complexity. Concomitant chronic dialysis or ventilator dependence were two factors that decreased the willingness of cancer patients to participate in DNR. Patients with chronic dialysis or ventilator dependence showed lower DNR rates than those suffering from non-major illnesses. Although chronic dialysis or ventilator-dependent patients suffer from incurable illnesses, their conditions are managed with LST and may seem stable. Doctors and patients may not have prepared for the end of life mentally, either. Our data might indicate the need to educate both patients and their family members to prepare for the deterioration of the patients’ state of health toward the end of life.

Our study demonstrated that the East region of Taiwan had the highest DNR rate. This region also had the lowest population density, the least number of large hospitals, and the least amount of medical resources (i.e., only one medical center). The relatively disadvantaged socioeconomic status in the East may have led to fewer life-sustaining treatments, thereby contributing to a higher DNR rate in this region[12]. On the other hand, the South region had the lowest DNR rate. The southern region is known to be more conservatively culturally. It may well be that less children in the southern Taiwan are willing to sign the DNR order for their parents because this may be viewed as not fulfilling filial duties[12]. Further qualitative studies are needed to understand whether special cultural beliefs (e.g., more Taiwanese indigenous peoples in East region) affect DNR rates.

Futile therapies usually increase medical expenditure but do not necessarily increase the quality of care at the end of life. Our definition of DNR (no resuscitation prior to death) essentially implies less intensive care, which is largely futile, at the end of life. A study of eight hospitals in the US from 2002 to 2004 showed that palliative care patients who died had an adjusted net savings of US$4,908 in pharmacy, laboratory, and ICU costs in direct costs per admission, and US$374 in direct costs per day[13], compared with usual care patients. Zhang et al. conducted a study regarding health care costs specific to the last week of life of 603 cancer patients and reported savings of US$1,041 in patients who had end-of-life discussions with their doctors[14]. Another study at a secondary care hospital in Oman by Ahmad et al. showed that institutional DNR policies may help to reduce healthcare costs, and that each potential DNR patient cost an average of USD $1,958.90 in healthcare[15]. Ahmad et al.[15] further considered that potential futile CPR patients can be identified with careful assessment, and that resuscitation of these patients can be avoided. Although the above studies showed a clear reduction in the patients’ expenses, they were limited to restricted hospitals or illnesses. In the United States, one study conducted a retrospective cost analysis through date of death of home-based palliative care patients with continuous claims months before program enrollment[16]. The authors found a 64% cost divergence between palliative and controlled cohort in the last three months of life. However, the program of the study was developed within a single, integrated, multistate health system with a robustly functioning accountable care organization. Our study is the first to employ nationwide database analysis to elucidate the differences in NHI spending between DNR and non-DNR patients in the last one year of life. Compared to DNR patients, the spending on non-DNR patients varied considerably because their survival periods differed depending on the type of resuscitation and LST, making it difficult to establish a single baseline to determine their NHI spending. To overcome these discrepancies, we approximately calculated all inpatient, emergency, and outpatient visits of DNR and non-DNR patients one year prior to death. Results confirmed that the NHI spending of the DNR group was significantly lower than that of the non-DNR group during this period. The DNR patients consumed from 7.48% to 12.33% less NHI spending than non-DNR patients. After adjusting for age, gender, calendar year and geographic areas, we found the estimated cost difference increased between DNR and non-DNR groups. As shown in Table 2, patients with dialysis were less likely to have DNR orders, and they were associated with substantial health expenditure. Had they had DNR orders, the cost savings associated with DNR would have been larger, as shown in the multivariable analysis. By promoting better end-of-life care and DNR, a larger portion of resources can be allocated to others who need health care most.

This study has some limitations. First, we could only identify the resuscitative procedures that were applied to patients before their deaths and could not confirm the DNR consent of each patient. The DNR rates reported in this study are represented by the percentage of patients who received no resuscitation before death. According to Taiwan’s laws, patients must receive life support measures unless they have DNR orders in advance. In other words, there are no other possible situations that patients did not receive life support measures other than they have DNR orders. Therefore, the non-resuscitation rate should reasonably approximate the DNR rate and this limitation should not materially affect the results. Second, we could only confirm if patients were suffering from cancer, but we could not determine if the cancer was terminal. The DNR behaviors of patients and their family members are closely associated with their illness and prognosis. Factors that influence cancer treatment and prognosis, such as cancer stage at the time of diagnosis and the patient’s willingness to cooperate, were not captured in this study.

In conclusion, our study suggests that DNR rates varied across cancer types and decreased among cancer patients with concomitant chronic dialysis or ventilator use. We also found disparities in DNR rates across geographic regions in Taiwan. A wider adoption of the DNR policy may achieve substantial savings in health expenses.
